# Responses of coral reef fishes to past climate changes are related to life‐history traits

**DOI:** 10.1002/ece3.2800

**Published:** 2017-02-26

**Authors:** Eduardo Ottimofiore, Camille Albouy, Fabien Leprieur, Patrice Descombes, Michel Kulbicki, David Mouillot, Valeriano Parravicini, Loïc Pellissier

**Affiliations:** ^1^ Unit of Ecology & EvolutionUniversity of FribourgFribourgSwitzerland; ^2^ Swiss Federal Research Institute WSLBirmensdorfSwitzerland; ^3^ Landscape EcologyInstitute of Terrestrial EcosystemsETH ZürichZürichSwitzerland; ^4^ IFREMER, unité Ecologie et Modèles pour l'Halieutiquerue de l'Ile d'Yeu, BP2110544311 Nantes cedex 3France; ^5^ UMR MARBEC (CNRS IRD IFREMER UM)Montpellier Cedex 5France; ^6^ Institut pour la Recherche en DéveloppementUR UMR “Entropie”, Labex Corail, Université de PerpignanPerpignanFrance; ^7^ CRIOBE, USR 3278 CNRS‐EPHE‐UPVD, LABEX “CORAIL”University of PerpignanPerpignanFrance

**Keywords:** climate change, dispersal, Indo‐Pacific Ocean, species distribution models

## Abstract

Coral reefs and their associated fauna are largely impacted by ongoing climate change. Unravelling species responses to past climatic variations might provide clues on the consequence of ongoing changes. Here, we tested the relationship between changes in sea surface temperature and sea levels during the Quaternary and present‐day distributions of coral reef fish species. We investigated whether species‐specific responses are associated with life‐history traits. We collected a database of coral reef fish distribution together with life‐history traits for the Indo‐Pacific Ocean. We ran species distribution models (SDMs) on 3,725 tropical reef fish species using contemporary environmental factors together with a variable describing isolation from stable coral reef areas during the Quaternary. We quantified the variance explained independently by isolation from stable areas in the SDMs and related it to a set of species traits including body size and mobility. The variance purely explained by isolation from stable coral reef areas on the distribution of extant coral reef fish species largely varied across species. We observed a triangular relationship between the contribution of isolation from stable areas in the SDMs and body size. Species, whose distribution is more associated with historical changes, occurred predominantly in the Indo‐Australian archipelago, where the mean size of fish assemblages is the lowest. Our results suggest that the legacy of habitat changes of the Quaternary is still detectable in the extant distribution of many fish species, especially those with small body size and the most sedentary. Because they were the least able to colonize distant habitats in the past, fish species with smaller body size might have the most pronounced lags in tracking ongoing climate change.

## 1 Introduction

Marine species are responding to climate change by tracking their environmental niche geographically (Chen, Hill, Ohlemüller, Roy, & Thomas, 2011; Hoegh‐Guldberg & Bruno, 2010; Parmesan, 2006). Species are characterized by an ensemble of life‐history traits, which shape their ecological preferences (Pörtner, Schulte, Wood, & Schiemer, 2010), but also how they respond to ecological perturbations (Mouillot, Bellwood, et al., 2013). Identifying life‐history traits that are indicators of species responses to climate change would allow to forecast the future of marine species assemblages and their supply of ecosystem services (Worm et al., 2006). Focusing on contemporary climate change, Sunday et al. (2015) evaluated the species life‐history traits associated with range expansions during the last decades and revealed a major role of fish swimming ability and ecological generalism. The Quaternary period was also characterized by rapid fluctuations of global temperatures (Lea, Pak, & Spero, 2000), which caused substantial species range shifts (Svenning & Skov, 2007). Quantifying dispersal lag from the Quaternary glaciation in species contemporary range could reveal the relevant life‐history traits modulating species responses to climate change (Lenoir & Svenning, 2014). Modelling the relationship between the geographic distribution of climate changes of the Quaternary and the extant distribution of species offers the opportunity to better evaluate lags of species responses to past climate changes and their capacity to track favorable environmental conditions (Sandel et al., 2011; Svenning & Sandel, 2013).

The current geographic distribution of both terrestrial and marine species is associated with contemporary environmental conditions including climate, light, or chemical properties (Barbosa & Schneck, 2015; Chuine, 2010). However, extant environmental conditions do not fully explain the distribution of species and additional variables should be considered, such as those linked to past environmental conditions (Svenning & Sandel, 2013). An increasing number of studies have documented the legacy of Quaternary climate change on the geographic distribution of species, for instance in trees (Svenning & Sandel, 2013), terrestrial animals (Carnaval, Hickerson, Haddad, Rodrigues, & Moritz, 2009; Sandel et al., 2011), freshwater (Leprieur et al., 2011), and marine fauna (Pellissier et al., 2014). Since the beginning of the glaciation period (ca. 2.58 million years ago), global climate variation followed multiple successive cycles of glacial and interglacial events, which gave rise to cycles of species range contractions and expansions (Bennett & Provan, 2008; Magoulick & Kobza, 2003; Svenning & Skov, 2007). Climate change drove shifts in the distribution range of species by hundreds of kilometers (Yannic et al., 2014). However, because the dispersal abilities of species are generally limited, most species were not able to recolonize their entire suitable habitat and display migration lags (Svenning & Sandel, 2013).

Variable degree of migration lags exists in extant species distribution (Lenoir & Svenning, 2014), which might relate to the species abilities to track climate change (Svenning & Sandel, 2013). Because of variable dispersal abilities, species are not always able to track climate change. As a consequence, the current geographic distribution of species might be more restricted than the suitable habitat available in the landscape (Chen et al., 2011; Dullinger et al., 2012; Svenning & Sandel, 2013). Lags under climate change is expected to be largely associated with species life‐history traits, as shown for terrestrial vertebrates with contrasted dispersal abilities (Sandel et al., 2011). By comparing the degree of migration lag among species to species traits, it may be possible to identify an indicator of species responses also designated as “response traits” (Lavorel & Garnier, 2002; Suding et al., 2008). Response traits have been typically used to understand how species respond to environmental changes (Lavorel & Garnier, 2002), in order to extrapolate over entire species pools. For instance, Foden et al. (2013) investigated which traits are associated with species vulnerability under climate change for birds, corals, and amphibians, and used the results to prioritize critical geographic areas for conservation. Sunday et al. (2015) evaluated the species life‐history traits modulating range expansions under climate and showed a compound role of swimming ability and ecological specialization. The advantage of studying species lags under past climate changes over ongoing changes is that species display large variance of lags (Lenoir & Svenning, 2014). If species with a set of traits failed to track past climate changes across millennia, responding to ongoing climate changes is expected to be even more challenging.

Coral reefs occupy less than 0.1% of the global ocean surface, but host a disproportionately large marine biodiversity (Pandolfi, Connolly, Marshall, & Cohen, 2011) by providing a habitat for at least 25% of all marine species (Spalding & Grenfell, 1997). While coral are distributed worldwide, reef‐forming species are constrained by temperature and light (Coles & Fadlallah, 1991; Coles & Jokiel, 1977; Hughes et al., 2012; Kleypas, McManus, & Meñez, 1999; Marshall & Clode, 2004), because of an association with photosynthetic microalgal endosymbionts called zooxanthellae. (Baker, Glynn, & Riegl, 2008; Hoegh‐Guldberg, 1999). As a result, coral reefs only occupy shallow waters in tropical oceans. Coral growth generates structurally complex reefs that provide habitat to many marine species (Pratchett, Hoey, & Wilson, 2014). Coral reefs provide shelter to fishes, and some fish species even feed on coral (Cole, Pratchett, & Jones, 2008). During the Quaternary, climatic fluctuations caused important changes in sea surface temperature (SST) and sea level (SL), which largely impacted the distribution of coral reefs (Pellissier et al., 2014). While conditions for coral growth likely degraded during glacial episodes along the coast of Northeast Africa or in the Pacific Ocean, other regions such as the Indo‐Australian archipelago remained stable and provided refugia for many species (Pellissier et al., 2014). Because of their trophic and habitat associations, climate change effect on coral reef distribution should in turn have impacted fish assemblages (Pellissier et al., 2014). Yet, the response of fish species to historical habitat changes should depend on species life‐history traits, such as dispersal abilities, or habitat specialization.

In this study, we quantified the lag of Indo‐Pacific coral reef fish in recolonizing extant suitable habitats after the glaciations of the Quaternary and tested the relationship with life‐history traits. First, we ran species distribution models (SDMs) relating the presence and absence of fish species to current environmental variables. We also supplied the models with predictors representing the distance to persisting habitats during Quaternary climate change. We quantified the relative importance of extant ecological conditions versus distance to stable areas during the Quaternary in explaining species distribution. This approach has been applied to trees in Europe (Normand et al., 2011), but was never applied to the fish assemblages of the Indo‐Pacific Ocean. In addition, while the role of the glaciations of the Quaternary on fish species richness has been evaluated (Pellissier et al., 2014), the variability of responses among species has never been quantified so far. Second, we related the variance purely explained by the historical variables to fish size, a categorical trait of mobility, and habitat specialization obtained from the literature (Descombes et al., 2015; Parravicini et al., 2014). Fish size, which is associated with both larval and adult dispersal abilities (Luiz et al., 2015), together with mobility should directly relate to species lag under climate change and would inform on the sensitivity of species to ongoing climate change.

## 2 Materials and Methods

### 2.1 Paleoclimatic data

We compiled paleoclimate data including SST and SL from ocean sediment cores archived in the National Climatic Data Center database for paleoclimatology of the National Oceanic and Atmospheric Administration. Many sediment cores have been extracted in the context of climate research program and record SST or SL at multiple locations (e.g., Miller et al., 2005). We selected sediment cores in the tropical to subtropical Indo‐Pacific Ocean situated between latitudes of 35°N and 35°S (Figs S1 and S2). We retained data sets starting before the Holocene (i.e., 15 ka before present, BP) and covering at least partially the Quaternary. We assembled 25 time series at a temporal resolution of 1,000 years. We performed Pearson correlations between time series to check for consistency.

We combined the SL time series from Miller et al. (2005) with a bathymetry model at 0.5° spatial resolution to map cells that are immerged at a temporal resolution of 1,000 years. We combined the 25 time series with a map of SST for the early industrial period, when the effect of anthropic climate changes were limited (1948–1958). This map was obtained by exploiting a global ocean hindcast simulation covering the prewarming decades of interest 1948–1968, coupling ocean‐sea ice module NEMO‐LIM in the configuration embedded in the EC‐Earth climate model (Sterl et al., 2012). We computed the temperature anomalies, representing the difference between the past SSTs reconstructed from the cores and the corresponding cell of prewarming temperature map. We calculated the average anomaly for each 1,000‐year time period across all cores and applied those anomalies to the preindustrial temperature maps. We obtained an ensemble of maps of immerged cells and paleotemperature for each 1,000 years.

We computed an environmental envelope, representing the coral reef habitat at a resolution of 0.5°, by overlaying favorable SST and SL maps. While the minimum mean annual temperature for the establishment of coral is expected to be situated around 25° with an optimum at approximately 26–27°C (Kleypas et al., 1999), we performed a sensitivity analysis on the temperature threshold. We computed habitat maps for each time steps using six different temperature threshold values from 23 to 28°C by 1°C increment. From these, we computed a variable representing the isolation from stable areas. We calculated the cumulative distance between a given cell and the closest cell suitable for coral reefs over the three million years divided by the number of times the cell was suitable over the same period (Pellissier et al., 2014). The lowest values represent area in proximity to stable areas while the highest represent locations with frequent local habitat collapse during the glacial periods. To compute geographic distances, we used the “costdistance” function in the package gdistance in R, to account for land masses as barriers to dispersal.

### 2.2 Fish geographic distribution data

Fish distribution information was extracted from about 500 references, covering 169 different locations, and then completed by the construction of the extent of occurrence map for each species (Parravicini et al., 2013; Pellissier et al., 2014). We defined the extent of occurrence as the convex hull polygon of locations where each species was present, where each polygon was individually inspected in comparison with knowledge of reef fish species distribution. In order to avoid discontinuities to be merged, polygons were separated in multiple polygons when necessary. Cells containing at least 90% of land, those without coral reef habitat, and those with an original gap of information (few cells in northwestern Australia) were excluded. The data were set at 5° resolution (i.e., ca. 555 km at the equator). The resulting distribution maps were validated by experts and were used in several studies on marine biodiversity patterns (Leprieur et al., 2016; Mellin et al., 2016; Pellissier et al., 2014). After geographically selecting the species from the Indo‐Pacific (5,263 species), we removed species with less than 10 cells of presence (1538 sepcies). We also excluded 161 species, whose SDMs did not converge.

### 2.3 Contemporary environmental predictors

We selected current annual mean SST, mean chlorophyll *a* (CHL), dissolved oxygen (O_2_), nitrate (NI), and sea surface salinity (SSS) as present condition variables, which were based on the Bio‐ORACLE data set (Tyberghein et al., 2012). SST and SSS can affect coral reef distribution, hence the habitat of fishes, but can also influence directly fish species distribution (Bowden et al., 2014; Donelson, Munday, Mccormick, & Nilsson, 2011; Sunday, Bates, & Dulvy, 2012). In addition, dissolved oxygen, mean CHL *a* levels, and nitrate concentrations can influence reef productivity and the composition of fish assemblages (Marubini & Davies, 1996). The environmental variables were aggregated at the cell resolution of the species distribution resulting in the 280 grid cells.

### 2.4 Species distribution models

We modelled the distribution of species using generalized linear models (GLMs), with linear and quadratic terms and a logit link function. This statistical technique is standard and commonly used in species distribution modelling (Calabrese, Certain, Kraan, & Dormann, 2014). The advantage of GLM over other modelling approaches is that the variance can be partitioned among predictors, which facilitates the ecological interpretation of distribution–environment relationships (Borcard, Legendre, & Drapeau, 1992). Estimation of parameters from most modelling techniques might be biased due to spatial autocorrelation (Swanson, Dobrowski, Finley, Thorne, & Schwartz, 2013). To evaluate whether our results are sensitive to this issue, we quantified the spatial autocorrelation in the residuals of the models using the Moran's I statistic. The final formula of the GLM model to obtain the probability of the presence of a species (*P* ^pres^) is the following:

$$\begin{array}{cc} \log\text{it}(P^{\mathrm{pres}})= & \alpha +\beta_{1,1}\text{SST}+\beta_{1,2}{\text{SST}}^{2}+\beta_{2,1}\text{CHL}+\beta_{2,2}{\text{CHL}}^{2}+\beta_{3,1}O_{2}\\ & +\beta_{3,2}O_{2}^{2}+\beta_{4,1}\text{NI}+\beta_{4,2}{\text{NI}}^{2}+\beta_{5,1}\text{SSS}+\beta_{5,2}{\text{SSS}}^{2}+\beta_{6,1}\text{IREF}\\ & +\beta_{6,2}{\text{IREF}}^{2} \end{array}.$$

We calculated the explained variance of the model (*D* ^2^) with the formula: *D* ^2^ = 1 − (SS_res_/SS_tot_), SS_res_ being residual deviance (residual sum of squares) of the models, and SS_tot_ the null deviance (total sum of squares). We quantified the explained variance of the model of each species with and without the historical variable.

We performed a variance partitioning to isolate the independent contribution of contemporary and historical variables as described in Borcard et al. (1992). This approach allows variance partitioning into four fractions: (1) pure contemporary variables; (2) shared contemporary and past; (3) pure past; and (4) unexplained variance (Borcard et al., 1992). Finally, we computed the area under the curves (AUCs) of the models when the historical predictors from the different temperature thresholds are added to the models. To compute AUC, we used 100× repeated split‐samples (10×) calibrating the model on 70% of the data and validating on the remaining 30%.

### 2.5 Biological traits data

We investigated the relationships between the variance purely explained by isolation from stable areas in the model of each species and the corresponding life‐history traits. We investigated body size, mobility, and habitat specialization. Maximum size of each species and home range size were extracted from the FishBase database (Froese & Pauly, 2016). The home range size trait was coded using three ordered categories: sedentary (including territorial species), mobile within a reef, and mobile between reefs. We compiled a large literature on the interaction of fish species with the coral reef to produce a trait of habitat specialization. This trait is classified into three categories, if a fish species is specialized on coral reefs, or not, or show a specialized relationship with another organism living in or in proximity to the coral reef habitat (e.g., algae, anemones, echinoderms, gorgonians, mangrove roots, soft corals, sponges). Finally, trait values for adult life stage were extracted from specific literature for the Indo‐Pacific (see Mouillot et al., 2013), the Atlantic (Halpern & Floeter, 2008) and from FishBase.

We related the variance purely explained by the historical variable in the SDMs and the corresponding life‐history traits using a linear model. We compared the Akaike information criterion (AIC) resulting from the model including different combinations of traits (Burnham & Anderson, 2002) using the “MuMIn” package (Bartoń, 2015). From all subset models, the Akaike weight (*w*) provides the probability that a specific model is the best among the others (Kissling, Field, & Böhning‐Gaese, 2008; Pellissier et al., 2014). By summing the Akaike weights (*w*_AIC_) of all models containing a specific trait, we obtained the relative importance of each trait (Burnham & Anderson, 2002; Diniz‐Filho, Rangel, & Bini, 2008; Sandel et al., 2011). We applied the phylogenetic generalized least squares (PGLS) method to test whether the relationship between traits and history remains significant once the phylogenetic relationships between species were accounted for in the analysis. We used the time‐calibrated phylogeny of Rabosky et al. (2013) which contains the relationship among 7,822 fish species worldwide. After removing species not associated with coral reefs, we obtained a phylogeny of coral reef fishes containing 2,310 species. We grafted the missing species‐based taxonomic information from fish identification guides and FishBase (Froese & Pauly, 2016). Specifically, new tips representing unsampled species were added to congeneric species. These analyses were performed in R (R Core Team 2015) with the “caper” package (Orme et al., 2013).

### 2.6 Mapping the importance of isolation from stable areas

We visualized the geographic patterns of the strength of the historical variable in the SDM by mapping the average contribution of the historical variable of all species occupying each cell. In addition, for comparison, we computed a map of the average fish size among the species occupying each cell. We related the average historical contribution within each cell related to the average fish size in each cell using a linear model and tested the significance of the relationship.

## 3 Results

### 3.1 Tropical reef distribution and thresholds comparisons

**Figure 1:**
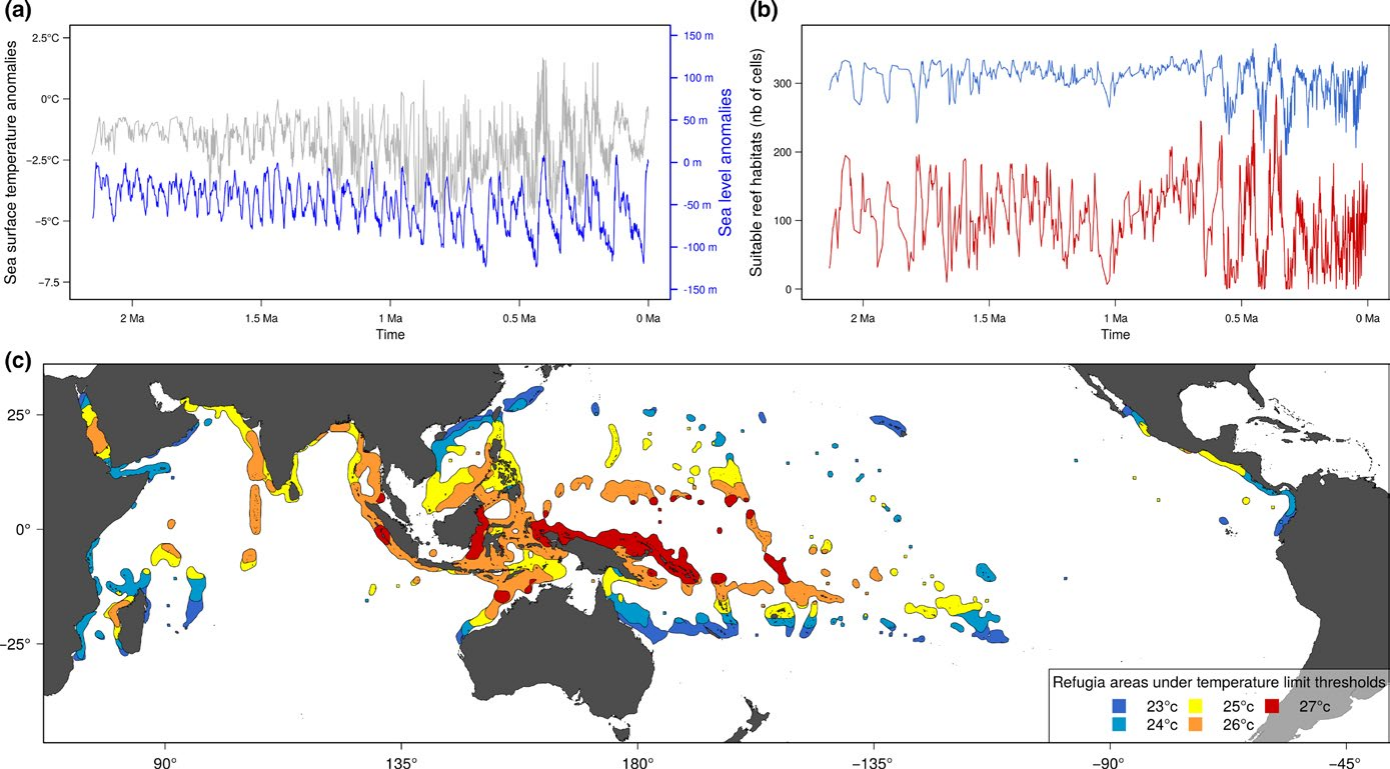
(a) Change in sea surface temperature (gray line) and sea level (blue line) through time. (b) Potential suitable reef habitat under two different temperature thresholds (blue line for 23°C; red line for 27°C). (c) Hindcasted tropical reef distribution in the Indo‐Pacific Ocean during the last glacial maximum (21 ka) based on sea‐level changes and also considering different temperature thresholds from 23 to 27°C

**Figure 2:**
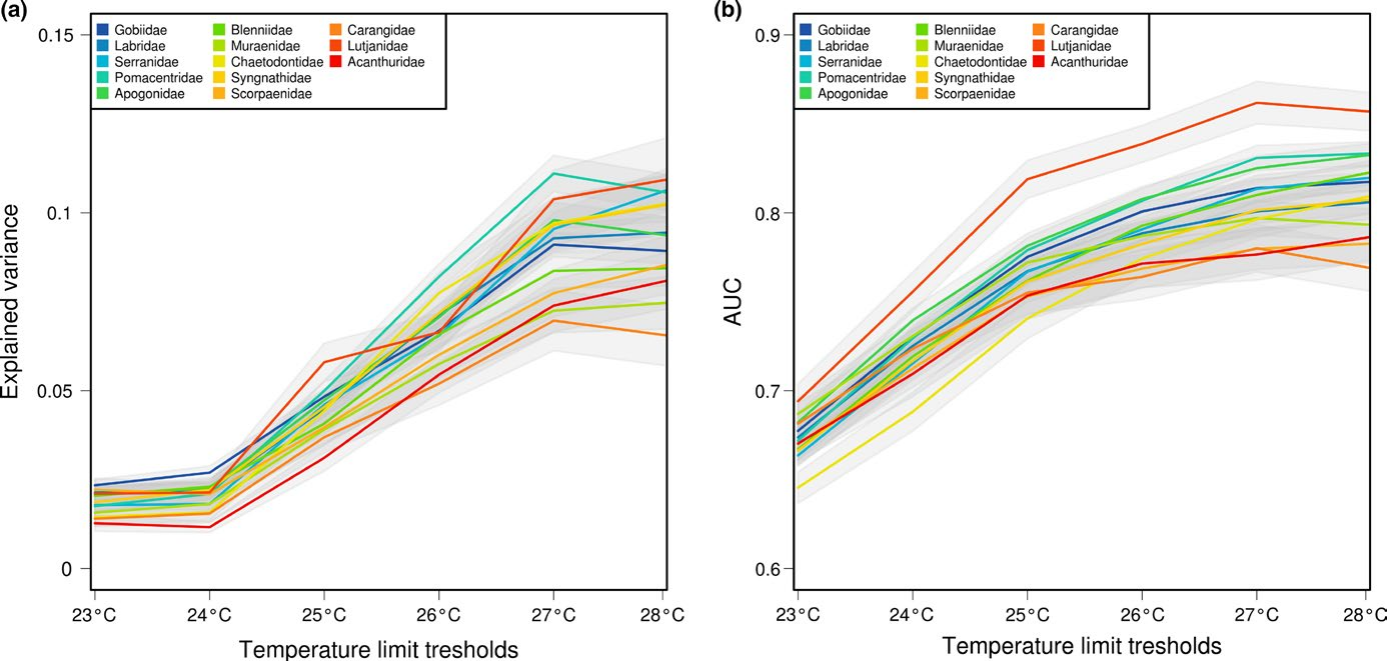
(a) Explained variance by the species distribution models containing only the historical variable for the main fish families and for the temperature threshold considered, (b) AUC of the historical model for the main fish families for the temperature threshold considered. Both AUC and explained variance showed a peak with a temperature threshold of 27°C

The distribution of the hindcasted potential coral reef areas depended on the temperature thresholds considered (Figure 1). The values of isolation from refugia varied according to the six temperature thresholds, but nevertheless shared similar general spatial patterns. In proximity to the Indo‐Australian archipelago, values of isolation from refugia are lower, while the highest values are distributed either in the eastern Pacific or at higher latitudes. We found that for most families, the variable of isolation from stable areas showed an increase in explanatory power beyond a threshold of 25°C (Figure 2a), with a higher median of explained variance with a threshold of 27°C. In contrast, below 25°C the distance to stable areas had a low contribution to the SDM. The AUC of the model showed a maximum with the 27°C temperature threshold (Figure 2b). The residuals of the models showed low spatial autocorrelation in the data set for all temperature thresholds (mean of Moran's *I* = 0.19), which is unlikely to bias the estimation of parameters of the models.

The full SDMs showed high explained variance (full model: mean *R* ^2^ = .53). When considering the contemporary variables only, the total explained variance was lower (mean *R* ^2^ = .48). Considering a 27°C threshold, adding the isolation from stable areas in addition to the current environmental conditions improved the explained variance of the models of most species. A total of 66% of species had at least 10% of variance improvement when the historical variable is included and 16.6% had a 40% of variance improvement. Part of the variance was purely explained by isolation from stable areas (mean *R* ^2^ = .09), but this portion largely varied among species from low (5th percentile: *R* ^2^ = .024) to high (95th percentile: *R* ^2^ = .21). Some species displayed a large explained deviance of the isolation from stable areas, for instance the Golden damselfish *Amblyglyphidodon aureus* (*R* ^2^ = .31), the pink anemonefish *Amphiprion perideraion* (*R* ^2^ = .36), or the wavy‐lined blenny *Entomacrodus decussatus* (*R* ^2^ = .31). In contrast, other species showed a lower explained deviance of the historical variable, for instance the black spotted butterflyfish *Chaetodon nigroponctatus* (*R* ^2^ < .01), the star snapper *Lutjanus stellatus* (*R* ^2^ < .01)*,* or the striped boga *Boops lineatus* (*R* ^2^ < .01).

### 3.2 Biological traits correlates of the importance of history

**Figure 3:**
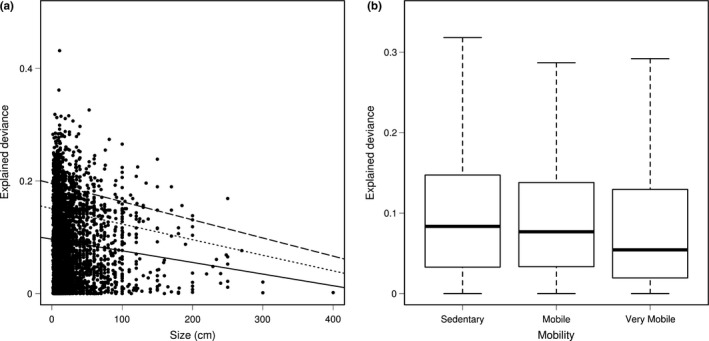
(a) Relationship between the species body size and the relative contribution of history in the species distribution models (SDMs). The lines indicate the linear regression (full line) and quantile regressions (*q* = .75, dashed; *q* = .9, long dashed) between the contribution of history and body size (cm) for tropical reef fish species considered in the study. (b) Relationship between species mobility and the relative contribution of history in the SDMs

We found a large variation in the variance purely explained by isolation from stable areas in the SDMs. We evaluated whether this variation was associated with life‐history traits. The independent variance contribution of isolation from stable habitats in SDMs was significantly related to life‐history traits in the statistical model. Summed Akaike's weights of all traits combinations showed that body size (*w*_AIC_ = 1; Table 1) and mobility (*w*_AIC_ = 0.90; Table 1) had a higher relative importance than association with reef habitat (*w*_AIC_ = 0.49; Table 1). Only body size and mobility were significant, but the slope was stronger for size than mobility. In addition, the PGLS analysis showed that body size was significantly correlated with the deviance explained by isolation from stable areas in the SDMs, independent of phylogenetic relationships among species (PGLS; λ = 0.114; *p* < .05). The relationship between size and the variance explained by isolation from stable areas was negative with larger fishes having the smallest historical contribution in the SDMs (Figure 3). This relationship was triangular, where small species showed the highest independent contribution of the isolation from stable areas but could also show a very low contribution. Similarly, the relationship between isolation from stable habitat and mobility remained significant after phylogenetic correction (λ = 0.1; *p* < .05). Sedentary species had a higher explained deviance than mobile and very mobile ones in the distribution models.

**Table 1:** Summary of the model averaging relating the effect of Quaternary history to life‐history traits

	Slope	*Z* value	*p*	*w*_AIC_
Size	−0.081	3.98	<.05	1
Mobility: sedentary	0.013	0.635	.53	0.9
Mobility: very mobile	−0.05	2.296	<.05	0.9
Habitat specialization: no	−0.04	1.94	<.1	0.49
Habitat specialization: specialized	−0.01	0.42	.67	0.49

The results of the minimum adequate model based on the OLS model is shown and retains all three variables. The relative importance of each predictor variable was assessed using the summed Akaike weights (*w*_AIC_).

### 3.3 Mapping the importance of isolation from stable habitat

**Figure 4:**
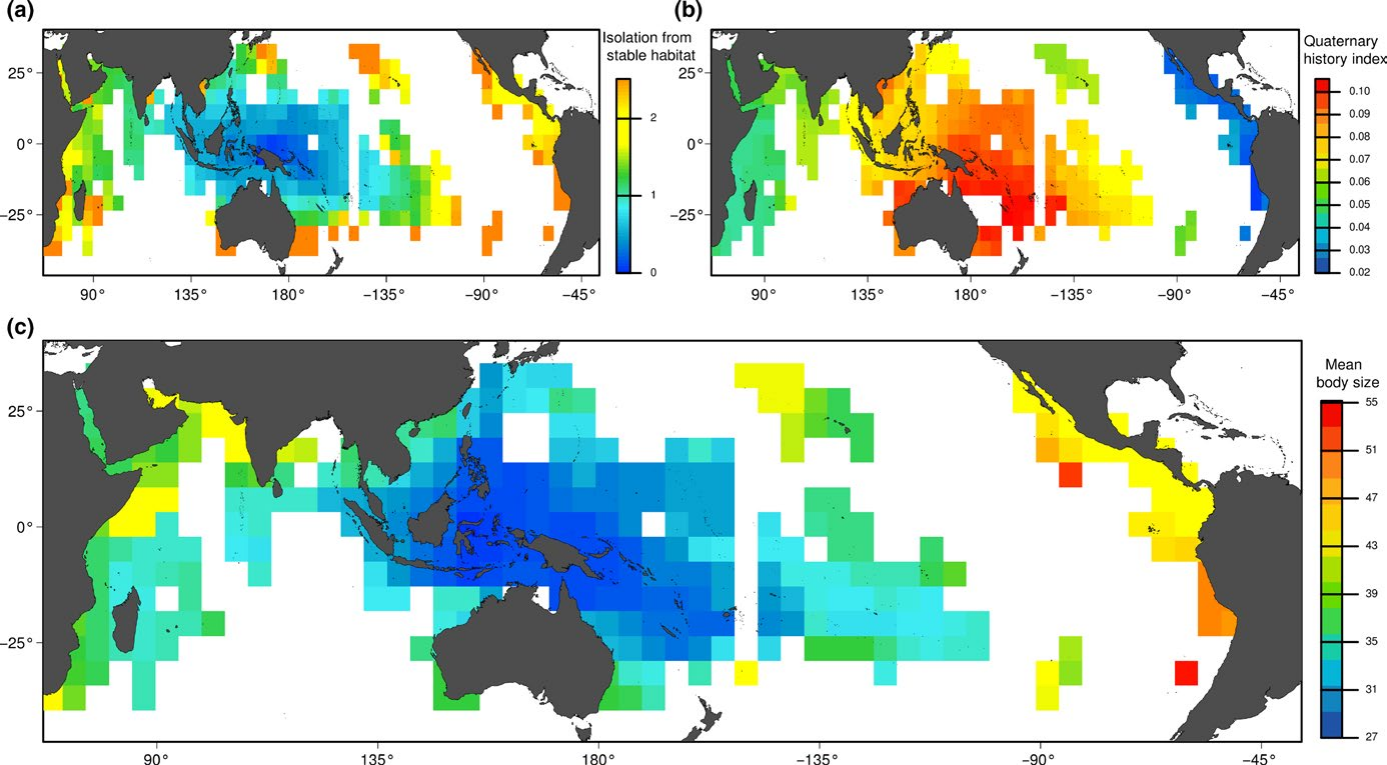
Maps of (a) isolation from stable habitat for the temperature threshold of 27°C; (b) mean independent explanatory power of Quaternary history in the species distribution models for a temperature threshold of 27°C; (c) mean body size (cm) per 5°× 5° cell across the Indo‐Pacific ocean of the species present in each cell. In the IAA, species assemblages are on average composed of smaller fishes and of species with a larger contribution of history in the models and therefore where current climate was not sufficient to explain their distribution

The spatial distribution of the mean contribution of isolation from stable habitat of each species in each cell showed patterned geographic distribution. With a temperature threshold of 27°C, the mean variance of the species present in each cell peaks in the Indo‐Australian archipelago (Figure 4a). We found that the mean body size (Figure 4c) within each cell showed the same spatial pattern than the average contribution of the Quaternary historical variable (Figure 4b) with a strong correlation with the mean explained deviance of the cells (*R* ^2^ = .66, *t* = −22.31, *p* < .001). Cells in proximity to the Indo‐Australian archipelago contained species whose SDMs showed a higher relative contribution of isolation from stable habitats. Those same cells corresponded to fish assemblages with the lowest mean size.

## 4 Discussion

Several studies illustrated the role of the Quaternary glaciation in shaping the extant distribution of species (Normand et al., 2011; Svenning & Skov, 2007). The quantification of the legacy of the climate changes of the Quaternary on current species distribution has been mostly carried out for terrestrial ecosystem, such as plants (Dullinger et al., 2012; Estrada et al., 2015) or mammals (Schloss, Nunez, & Lawler, 2012). In contrast, lags in species response to past climate change is poorly documented for the marine realm. Here, we show that supplying the SDM of fish species with a variable representing isolation from stable coral reef habitats during the Quaternary improves the variance of SDMs. Following previous studies relating the degree of lag to species life‐history traits (Estrada et al., 2015), we show that maximum fish size and mobility are good correlates of species lags. Our study, pointing out that species with specific traits are less likely to track climate changes, agrees with recent observations under ongoing climate change (Sunday et al., 2015).

The climate of the Quaternary comprised up to 50 pronounced glacial–interglacial cycles (Lambeck, Esat, & Potter, 2002; Zachos, Pagani, Sloan, Thomas, & Billups, 2001), which impacted the distribution of coral reefs directly via SST or indirectly through sea‐level change. The distribution of coral reef habitats largely changed through time during the Quaternary (Kleypas, 1997). During the most recent glacial period, the available surface might have been reduced by up to 88% (Kleypas, 1997). Our habitat reconstructions through the Quaternary showed strong temporal fluctuations associated with climate change oscillations. Temperature changes likely caused a contraction of coral reefs, especially in the eastern and western margins of the Indo‐Pacific Ocean (Pellissier et al., 2014). In addition, the amplitudes of sea‐level change during the Quaternary ranged from 60 to over 100 m (Figure 1b) during the strongest glacial episodes (Rohling et al., 2014). Because coral reefs are confined to less than 100‐m water depth, sea‐level changes caused habitat shifts during low sea stands. Shift in the distribution of coral reefs should have in turn impacted the distribution of fishes. We found that the variable of isolation from stable areas showed a good explanatory power for most families but only beyond a temperature threshold of 25°C. Below this threshold, the contribution of the variable of isolation from stable areas was weak. This threshold corresponds well to previously documented temperature threshold for the formation of coral reefs, expected to be close to 25°C (Kleypas et al., 1999). Moreover, the explained deviance was highest with a temperature threshold of 27°C considered as the optimal temperature for the growth of coral reefs.

The explained variance of isolation from stable habitats in the SDM largely varied among species as found on terrestrial taxa (Estrada et al., 2015). The explained variance by isolation from refugia in the SDMs was associated with life‐history traits. Among the selected species traits, maximum fish body size had the strongest relationship with the variable of isolation from stable habitat. In addition, the distribution of smaller species spatially matches areas close to stable coral reef habitats during cold periods (Figure 4). In contrast, species with larger body size occur both in area close to stable areas and in more isolated patches including the tropical eastern Pacific. Stier, Hein, Parravicini, and Kulbicki (2014) attributed the observed body size gradient across the Pacific to the different colonization capacity of small versus large species, and our study suggests that this body size gradient might have been partially shaped by habitat shifts during the Quaternary. Larger fish species produce many gametes with a longer larval duration, are more mobile as adult (Luiz et al., 2015) with larger home range (Nash, Welsh, Graham, & Bellwood, 2015), and can thus more easily track suitable habitat under climate change. In contrast, smaller fishes produce few gametes, have a shorter larval duration, and are thus expected to be more dispersal limited. In addition, we found that fish mobility was also correlated with the variance explained by isolation from stable habitats in the SDMs. Sedentary species generally usually show poor dispersal abilities, because the evolution of specialization in reef fishes is associated with strong homing behavior (Feary, 2007). Our results parallel observations made for terrestrial systems, where the legacy of climate change varied according to life‐history traits (Baselga, 2012).

According to our results, we may expect that under ongoing climate change small fish species that are more sedentary might be less able to track shifting suitable conditions compared to larger ones. Currently, small fish species occur mainly at lower latitudes in warmer waters in proximity to stable areas during the Quaternary. Yet, these reefs are expected to be the first exposed to critical thermal stress under climate change (Descombes et al., 2015). In contrast to large fishes, small fishes are therefore less likely to colonize newly formed coral reefs at higher latitudes (Riegl & Piller, 2003). Our results parallel findings of studies focused on the response of species to ongoing climate change. Feary et al. (2014) showed with a meta‐analysis that tropical fish species with large body size, high swimming ability, large size at settlement, and pelagic spawning behavior are more likely to show successful settlement into higher latitude under ongoing climate change. Similarly, Sunday et al. (2015) showed in the reefs of Southeast Australia that increased dispersal capacity and ecological generalism promote range expansion under climate change. Hence, inferences from past climate changes tend to agree with observation of range shift under ongoing climate change: poor dispersers and habitat specialist are slower to track shifting suitable habitats under climate change. Nevertheless, the rate of evolution of smaller species might be higher, which might allow faster adaptation to new environmental conditions (Munday, Jones, Pratchett, & Williams, 2008).

As any study relying on paleoenvironmental reconstruction, our work presents some limitations. Because of the few numbers of sediment cores for spatial interpolation during the early Quaternary, we used the mean anomaly across the Indo‐Pacific Ocean to map past habitat changes. However, it is possible that regional differences between the Indian or Pacific ocean exist and we might have locally under‐ or overestimated the effect of Quaternary climate changes. Second, while process‐based hindcasted oceanographic models might provide more accurate local representation of Indo‐Pacific hydrography, those are generally available only for the last glacial maximum. Although our study could identify traits–environment interactions, the applicability of our results to future climate change should be cautious. In fact, any extrapolation would rely on the assumption that Quaternary climate change is comparable to ongoing climate change. However, the velocity of ongoing climate change is much higher than during the Quaternary. Even species that were able to track suitable habitat under past climate changes might not do so in the future.

Together, our results showed that body size and mobility were best correlated with the variance explained by isolation from stable areas in the SDMs. While large fishes currently occupy area distant from putative stable areas during the Quaternary, such as the tropical eastern Pacific from Quaternary, many small fish species remain in proximity to the Indo‐Australia Archipelago. Our study suggests an interaction between species life‐history traits, habitat history, and species distribution. Life‐history traits constrain the persistence and movement of species under habitat changes, which is expected in turn to shape the species diversity (Pellissier et al., 2014) and functional structure (Stier et al., 2014) over time across the oceans. In complement to our results, studies along oceanographic gradients should monitor the speed of species range shifts under climate change but also compare it to species traits, employing a integrated approach that is currently lacking (Sunday et al., 2015).

## Conflict of Interest

None declared.
