# Supplementary material for: Responses of coral reef fishes to past climate changes are related to life‐history traits
Source: Ecol Evol. 2017 Feb 26;7(6):1996–2005. doi: 10.1002/ece3.2800 (PMC5355194; doi:10.1002/ece3.2800)
Supplement: Supplementary file 1 [file ECE3-7-1996-s001.docx]

**Supplementary materials**

**
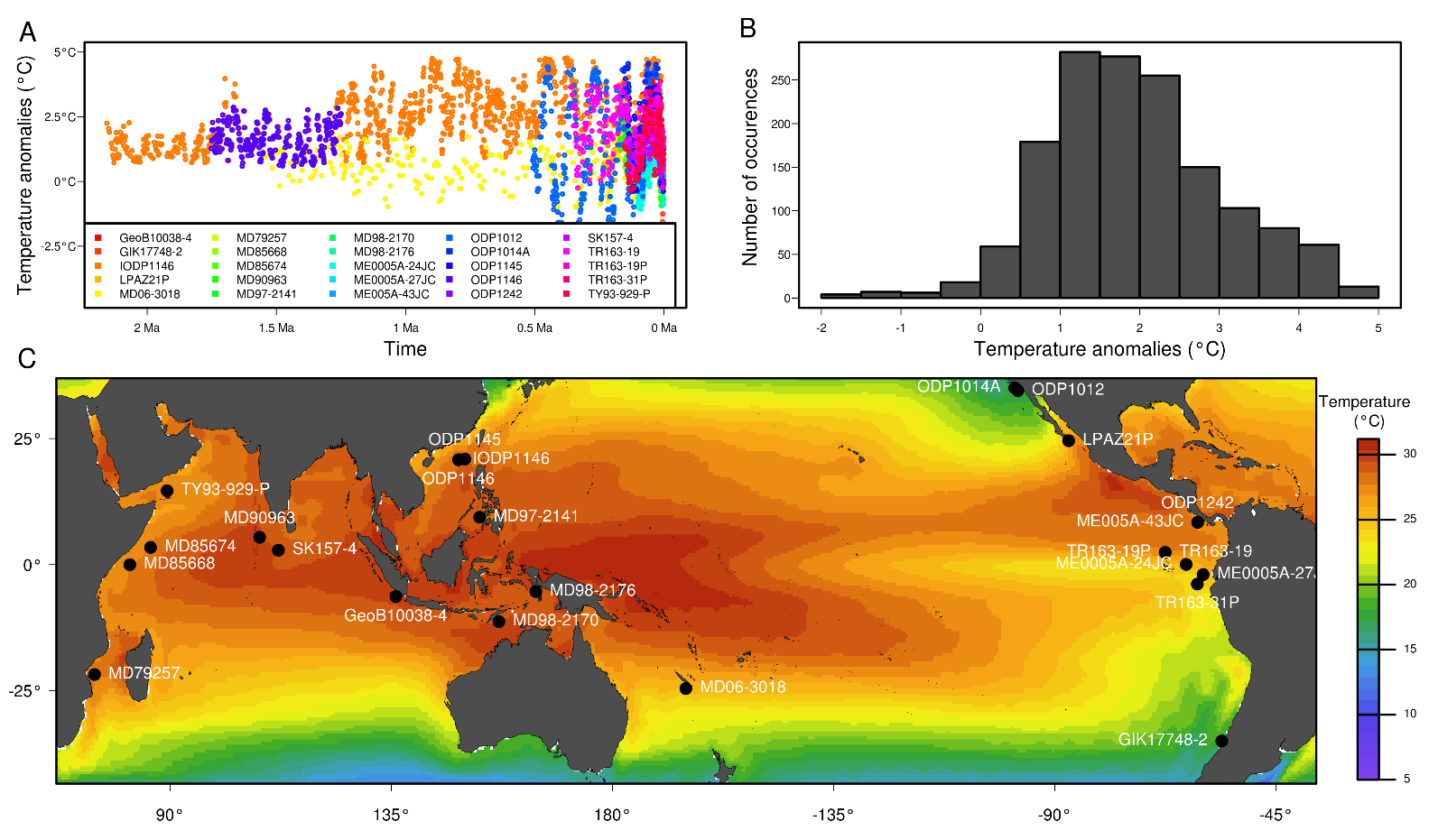
**

**Figure S1.** A. Temporal distribution of the sediment cores used to reconstruct the sea surface temperature across the Indo-Pacific Ocean. Note that in this graph positive anomalies correspond to temperatures colder than the present period. B. Histogram of the computed temperature anomalies, representing the difference between the pre-warming temperature map and the past SSTs reconstructed from the cores. C. Spatial distribution of the sediment cores used to reconstruct the variation of sea surface temperature across the Indo-Pacific Ocean. The map background represent the sea surface temperature for the pre-warming period, when the effect of anthropic climate changes were less marked (1948-1958).


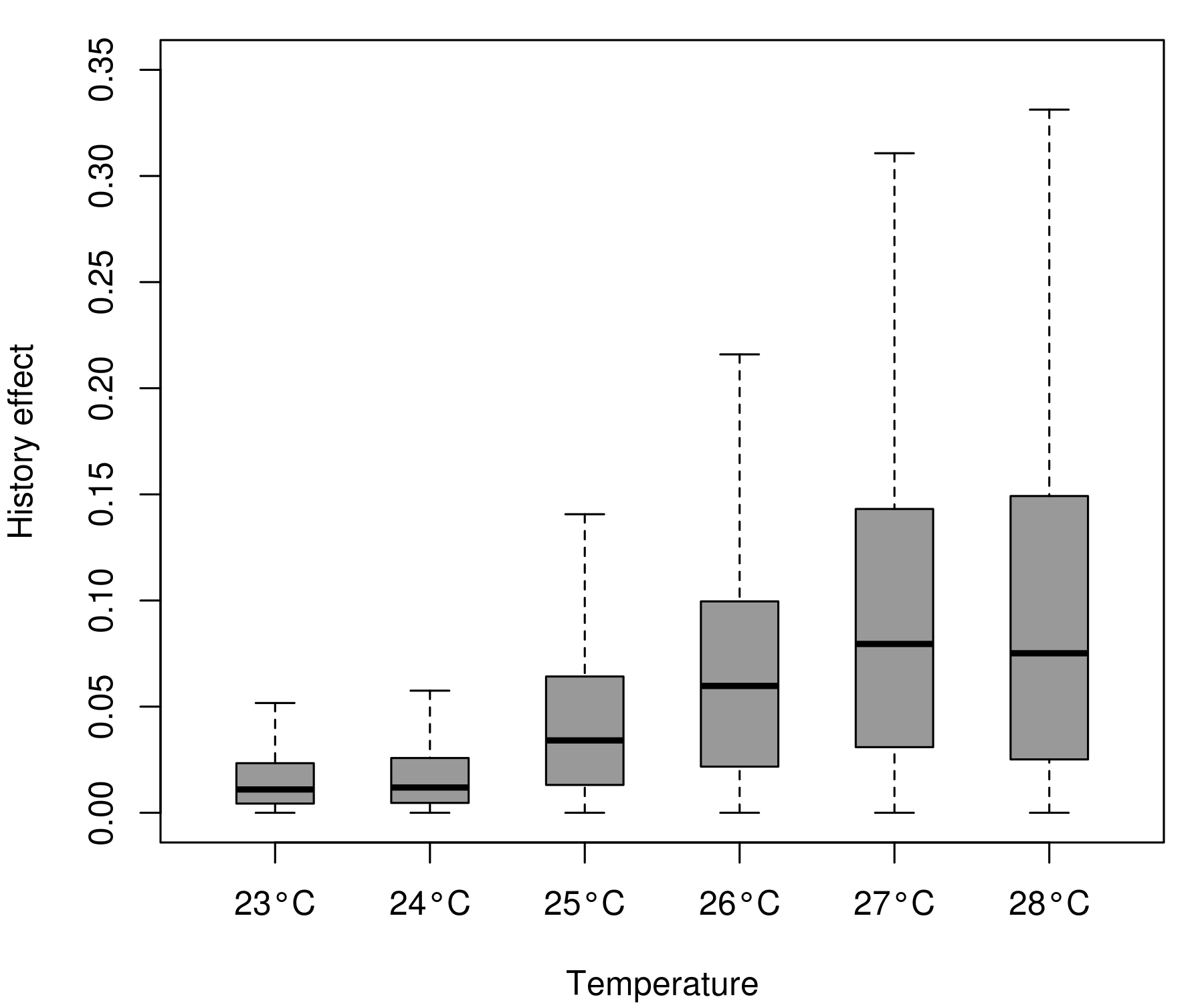


**Figure S3.** Boxplots of the variance explained independently by the historical variable computed using the different lower temperature thresholds from 23 to 28°C.


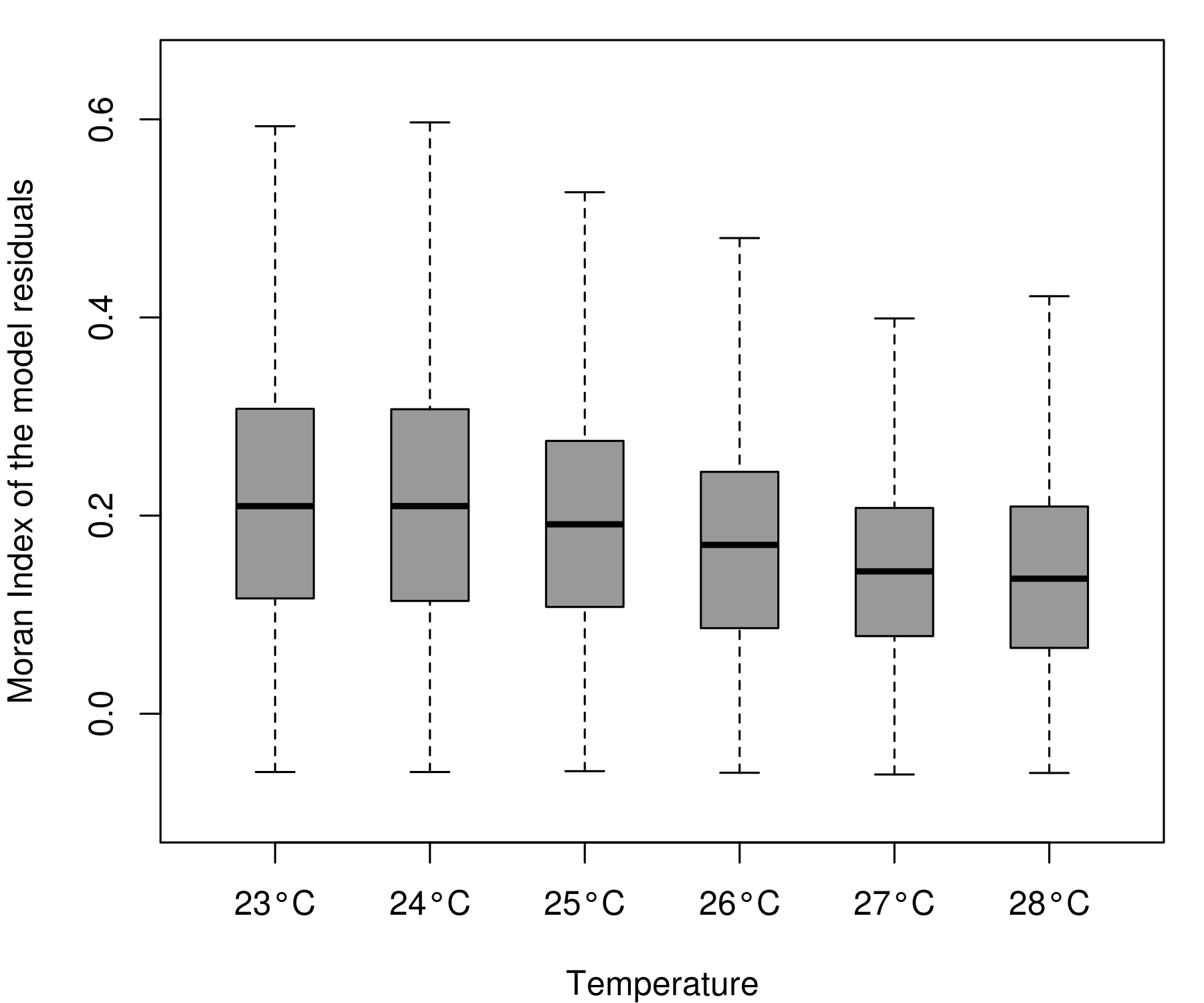
**Figure S4.** Boxplots of the Moran index of the model residuals computed using the different lower temperature thresholds from 23 to 28°C.

| Core ID | Publication |
| --- | --- |
| GeoB 10038-4 | Mohtadi et al. 2010 |
| GIK 17748-2 | Kim et al. 2003 |
| IODP1146 | Herbert et al. 2010 |
| LPAZ 21P | Herbert et al. 2001 |
| MD06-3018 | Russon et al. 2010 |
| MD79257 | Bard et al. 1997 |
| MD85668 | Bard et al. 1997 |
| MD85674 | Bard et al. 1997 |
| MD90963 | Bard et al. 1997 |
| MD97-2141 | Rosenthal et al. 2003 |
| MD98-2170 | Stott et al. 2007 |
| MD98-2176 | Stott et al. 2007 |
| ME0005A-24JC | Dubois et al. 2011 |
| ME0005A-27JC | Dubois et al. 2011 |
| ME005A-43JC | Benway et al. 2006 |
| ODP1012 | Herbert et al. 2001 |
| ODP1014A | Yamamoto et al. 2007 |
| ODP1145 | Oppo et al. 2005 |
| ODP1146 | Cleaveland et al. 2007 |
| ODP1242 | Benway et al. 2006 |
| SK 157/4 | Saraswat et al. 2005 |
| TR163-19 | Lea 2004 |
| TR163-19P | Dubois et al. 2011 |
| TR163-31P | Dubois et al. 2011 |
| TY93-929/P | Bard et al. 1997 |

**Table S1.** Identity of the sediment cores considered in the study and associated references.
